# Cost-effectiveness of a pictorial medication sheet for older adults with heart failure and cognitive impairment

**DOI:** 10.1186/s12913-026-14072-6

**Published:** 2026-01-21

**Authors:** Kaylee B. Vannoy, Lee Ann Hawkins, James G. Kahn

**Affiliations:** 1 Institute for Global Health Sciences, University of California, San Francisco, San Francisco, CA USA; 2 Graduate Nursing Division, Indiana Wesleyan University, Marion, IN USA; 3 Philip R Lee Institute for Health Policy Studies, University of California, San Francisco, San Francisco, CA USA

**Keywords:** Heart failure, Cognitive impairment, Pictorial medication sheet, Cost-effective, Medication adherence

## Abstract

**Background:**

Older adults with chronic health conditions like heart failure (HF) and cognitive impairment face challenges managing complex medication regimens, leading to poor adherence, worsening clinical status, and increased healthcare costs. Many existing interventions to improve medication adherence depend on digital technologies that may be too complex or require consistent caregiver support limiting their effectiveness in this population. Prior research demonstrated the efficacy of a simple pictorial medication sheet intervention compared to usual care. This study evaluates the cost-effectiveness of a pictorial medication sheet compared to usual care for older adults with HF and cognitive impairment.

**Methods:**

We developed a decision tree model with disease states in Microsoft Excel to evaluate changes in costs and quality-adjusted life years (QALYs) after implementing the intervention for older adults living with cognitive impairment and HF. The base case model used a one-year time horizon with adherence pathways and clinical outcomes, including decompensation, recovery, worsened or stable HF, and death. QALYs were calculated based on survival pathways and changes in health state utility. Costs included the intervention’s implementation and direct medical expenses related to HF care in the US. Sensitivity analyses (one-way, scenario, and Monte Carlo) explored how uncertainty in parameter inputs and time frame affects results.

**Results:**

Over a one-year time horizon, our model showed individuals using the pictorial medication sheet intervention had a total cost of $22,557 and 0.691 QALYs. Compared to usual care, the intervention was dominant (less costly and more effective), with $1,949 lower cost and a gain of 0.0045 QALYs, thus implementation for 100 individuals would potentially yield 0.45 QALYs and save $195,000 annually. Sensitivity analyses found that the intervention remained dominant over one year across plausible parameter ranges. A two-year analysis found that the intervention remained dominant across all tested scenarios, with QALY gains increasing almost six times more than the one-year model and cost savings ranged from $2,279 to $4,497 per patient.

**Conclusions:**

The pictorial medication sheet appeared to be cost saving and more effective than usual care for older adults living with cognitive impairment and HF in the United States. This finding supports evaluating strategies to implement the pictorial medication sheet at scale.

**Clinical trial number:**

Not applicable.

**Supplementary Information:**

The online version contains supplementary material available at 10.1186/s12913-026-14072-6.

## Background

Adults aged 65 years and older face many challenges related to aging, including polypharmacy resulting from potentially needing to take an increasing number of medications to maintain health [1]. In particular, older adults living with chronic conditions such as heart failure (HF) often have multiple comorbid conditions and are asked to manage complex medication and lifestyle regimens [2]. Guidelines recommend 4–6 medications for HF alone, and with comorbidities, these patients are often prescribed 10 or more medications daily [3, 4]. Polypharmacy has been shown to be a major factor leading to poor adherence resulting in poorer clinical outcomes including frequent hospitalizations, high mortality, reduced quality of life, and increased healthcare costs [5–8].

Medication management is especially challenging for individuals with cognitive impairment, who tend to have chronic comorbid conditions such as HF [9]. The number of older adults with cognitive impairment is growing rapidly in the US, as longer life expectancy is associated with cognitive decline [10, 11]. In 2022, an estimated one-third of people 65 years old and older in the US live with cognitive impairment, and this proportion is likely to increase in the future without intervention [11, 12]. Cognitive impairment can lead to poor medication self-management because it interferes with memory and executive function, the ability to follow complex instructions [13]. This may lead to reliance on caregivers or the expectation for older adults to adapt to new technology, which can be confusing or inaccessible [14, 15]. Older persons living with HF and cognitive impairment are an at-risk population, and improving their medication adherence is a key factor in improving outcomes and reducing healthcare costs. Existing barriers to improving adherence illustrate the need for interventions that are simple and cost-effective to implement, simple to use, and tailored to cognitive limitations.

Behavioral economics offers promising ideas on how to effectively address the challenges of medication adherence. This field combines the principles of cognitive psychology and economics to explain how “biases” and habits can lead to apparently irrational or suboptimal choices [16, 17]. One important scenario is task complexity in the context of impaired cognitive attention or ability. In healthcare, behavioral economics is often applied through structural “nudges” that influence personal choices toward healthier behaviors [17]. Examples include default prescription refills or medication/vaccination reminders [17].

There are behavioral economic interventions that have been successful in improving medication adherence, including mobile apps, telehealth monitoring, telephone reminders, and automatic pill dispensers [18–21]. These interventions, though effective, have limitations in older adult populations because they require digital health literacy, access to interventions, and consistent support from a caregiver [22–24]. Thus, they may not be effective or sustainable long-term for older adults with cognitive impairment, many of whom have a lack of caregivers or social support. Few studies have evaluated behavioral economic intervention effectiveness for older adults with cognitive impairment.

One promising behavioral economic intervention for all older adults is the pictorial medication sheet. It is a visual aid for taking medications. It simplifies complex regimens by using clear, colorful images of pills with their names arrayed by the time of day of when they should be taken [14, 25]. This intervention demonstrated statistically significant improvement in medication adherence among older adults with HF and cognitive impairment, increasing average adherence from pre-intervention 79.7% to post-intervention 84.7% with an effect size of 0.42 [25]. The pictorial medication sheet was well received by participants with > 70% reporting that it helped manage their medication regimens [25]. The intervention’s cost and cost-effectiveness have not been evaluated [25].

Poor medication adherence for older US adults remains as a significant driver of hospitalizations, healthcare costs, and mortality [26]. The pictorial medication sheet may serve as a low-cost and scalable intervention to improve adherence. This cost-effectiveness study aims to assess the pictorial medication sheet health impact and costs compared to usual care. The analysis can inform stakeholders (patients, providers, and insurers) about the potential value of a practical tool to enhance medication adherence.

## Methods

### Overview

We developed a decision tree model incorporating disease states to simulate health and cost outcomes from the implementation of the pictorial medication sheet compared to usual care among older adults living with cognitive impairment and HF. The decision tree model presented one-year and partial year probabilities for clinical outcomes, utility values, and costs of people living with a HF diagnosis who are prescribed medications. Clinical outcomes included death, worsening HF symptoms (clinical exacerbation / decompensation), recovery, and maintaining stable HF. Each clinical state was assigned a health state utility (0–1), and each clinical path was translated into quality-adjusted life years (QALYs). Costs included direct medical costs such as implementation of the intervention, hospitalization, and medications. All input data were derived from literature. Sensitivity analyses used to assess how input parameter uncertainties affect results included one-way, scenario, and Monte Carlo simulations. The decision tree model was built in Excel. This study evaluation followed the Consolidated Health Economic Evaluation Standards 2022 protocol (Table S7, see Additional File 1) [27].

### Intervention and study population

**Fig. 1:**
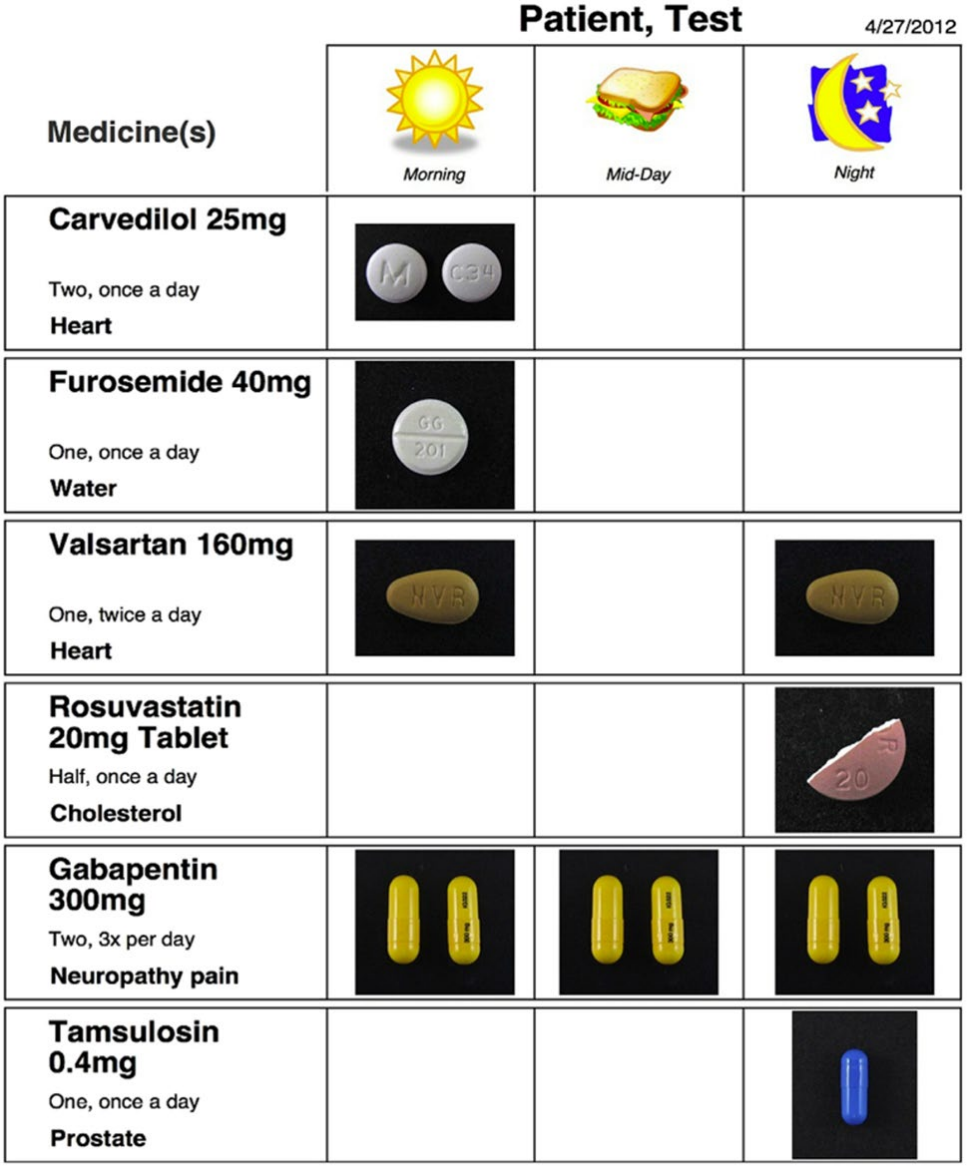
Example photo of pictorial medication sheet intervention. Reproduced with permission from Hawkins & Firek [25]. Heart & Lung 43 (6):486–493

This analysis was based on secondary data from the pictorial medication sheet intervention study, seen in Fig. 1, to improve medication adherence study [25]. The details from the Hawkins and Firek (2014) study that are most related to this analysis are described below.

Participants in the study were veterans living with HF and cognitive impairment enrolled from the outpatient VA HF clinic in southern California. All participants were recruited as a convenience sample during routine clinic visits throughout the study period, and the sample size was determined by feasibility during a fixed enrollment period. A total of 36 veterans were enrolled; 27 completed the protocol. All participants were independent adults living in the community who were managing their own complex medication regimens, taking five or more medications (for HF and other diagnoses) and adhering to multiple daily schedules. The mean Saint Louis University Mental Status (SLUMS) Examination score in the study population was 22 (SD ± 2.93), which places the participants in the mild cognitive impairment to upper range of dementia category; however, this score is only a screening tool and not a formal diagnosis. Patients with normal cognition, moderate-severe dementia, and caregiver dependent were not included. The study evaluated the pictorial medication sheet and its effectiveness in helping individuals follow medication instructions and improve adherence. The pictorial medication sheet included color images of each pill arrayed visually by the time of day the drug should be taken (morning, noon, evening), medication names, dosages, and indication stated in simple lay terminology. The intervention was designed to ease regimen adherence, especially for patients who recognize pills visually rather than by name. Adherence was measured using 30-day direct pill counts over a four-month period both before and after receiving the intervention [25]. The primary outcome was the change in medication adherence rates with potential for improved HF outcomes.

The comparator was standard medication management practices where providers offer medication instructions, but no adherence support aids are provided. This represents current real-world standard of care.

### Study perspective

Evaluation of cost-effectiveness was conducted from a societal perspective. The goal of this study was to inform US resource allocation decisions, specifically assessing the impact of implementation of pictorial medication sheets in hospitals and pharmacies. This perspective included direct healthcare costs for intervention implementation, primary and outpatient care visits, pharmacy visits, and long-term care. Costs reflected total allowed amounts, incorporating both insurer (e.g., Medicare) payments and patient cost-sharing.

### Time horizon

The time horizon for this analysis was one year because of the availability of published data for clinical outcomes and costs. A sensitivity analysis considered a two-year time horizon including uncertainty in long-term intervention efficacy and costs compared to usual care.

### Willingness-to-pay threshold

A willingness-to-pay threshold of $150,000 per QALY gained was used (for the few non-dominant results) to reflect a common benchmark for cost-effectiveness in the US [28].

### Model structure

**Fig. 2:**
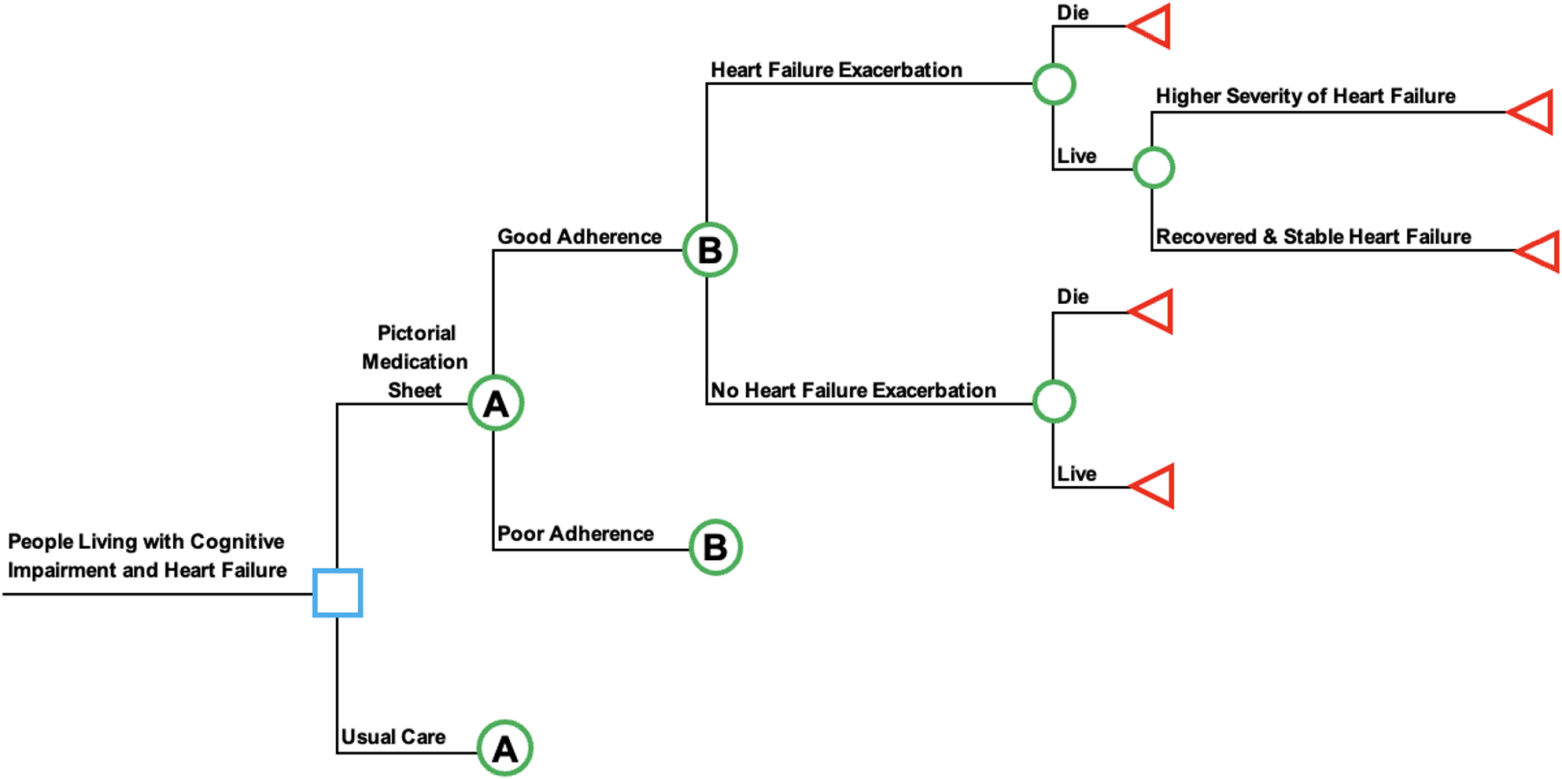
Decision tree model structure of people living with HF and cognitive impairment. The blue square is the initial decision between the pictorial medication sheet intervention and usual care. Green circles represent chance nodes on potential clinical pathways, and red triangles are final health outcomes. Letters A and B indicate identical downstream branches shared across the intervention and usual care

The patient age of 75 years old was used to reflect the mean age in the Hawkins & Firek (2014) study and to align with US population data showing the highest prevalence of HF among adults aged 75 years and older [29]. Figure 2 is a simplified version of the decision tree model comparing the pictorial medication sheet to usual care for people living with cognitive impairment and HF. The model specified health outcomes related to HF exacerbation (decompensation), recovery, recovered state, and mortality.

A decision tree model was chosen because the health outcomes are relatively simple, the time horizon for effects are relatively short, and there are clear decision and chance points. This type of modeling allowed explicit choices and incorporated probabilities for each of those chosen paths. In Fig. 2, the choice node (square) represents the decision between the intervention or usual care. The chance nodes (circles) portray potential clinical outcomes and paths. The decision tree ends with terminal nodes (triangles) which represent final health outcomes. Transitions between states are informed by published clinical probabilities.

The effectiveness of the pictorial medication sheet was assessed by comparing pre- and post- intervention adherence rates, categorized as poor (less than 80%) or good adherence (greater than or equal to 80%). The 80% cutoff was chosen based on empirical research on the association of adherence and clinical outcomes (discussed below). Clinical states in the model included death, worsening HF symptoms, recovery, and stable HF.

Total QALYs and costs for each path were calculated based on the events in the path and duration of specified states (e.g., time alive before death, or duration of healthier and sicker states). Model parameters were assigned appropriate probability distributions to reflect uncertainty. Clinical transition probabilities and health state utility values were modeled with beta distributions, with alpha and beta parameters derived from published mean values and 95% confidence intervals or assumed ± 25% ranges were unavailable (± 50% for the gain in adherence only) to ensure realistic curvature bounded between 0 and 1. All cost parameters were modeled using standard gamma distributions (symmetrical and asymmetrical), with shape and scale parameters calculated to match high and low values from literature sources or ± 25% assumed ranges when only single estimates were available. This approach allowed skewed, strictly positive cost distributions. Detailed parameter values, distributions, and sources were reported in detail in Table 1.

While cognitive impairment is an important health condition and factor in adherence, HF provides a clinically defined condition with measurable outcomes necessary for cost-effectiveness modeling. The decision tree itself is available from the first author. Model validity was assessed by checking internal logic and consistency in formulas and evaluating extensive one-way sensitivity analyses.

**Table 1:** Model parameters and ranges. Parameters and values were derived from published literature and adapted from multiple sources [25, 30–38]

Parameters	Base case	Range	Basis	Distribution	Source
Clinical Probabilities					
Good Adherence (Usual Care)	0.556	0.417–0.695	(± 25%)	Beta	[25]
Gain in Good Adherence (Intervention)	0.185	0.093–0.278	(± 50%)	Beta	[25]
HF Exacerbation with Good Adherence	0.232	0.174–0.289	(± 25%)	Beta	[30]
HF Exacerbation with Poor Adherence	0.419	0.314–0.524	(± 25%)	Beta	[30]
Live Following Exacerbation	0.597	0.448–0.747	(± 25%)	Beta	[31]
Increased Severity of Heart Failure	0.150	0.113–0.188	(± 25%)	Beta	[32]
Live Without Exacerbation	0.865	0.854–0.876	95% CI	Beta	[33]
Health State Utilities					
HF Exacerbation	0.560	0.555–0.565	95% CI	Beta	[34]
Stable HF	0.780	0.775–0.785	95% CI	Beta	[34]
NYHA II-IV Mean	0.600	0.565–0.635	95% CI	Beta	[35]
Costs					
Intervention Year 1	$ 498	$373 - $622	(± 25%)	Gamma	Suppl. Tables S1, S2, & S3^a^
Intervention Subsequent Year	$ 400	$ 300 - $ 500	(± 25%)	Gamma	Suppl. Table S2^a^
HF Hospitalization	$ 33,851	$ 6,603 -$ 73,382		Gamma	[36–40]
Outpatient Visit	$ 1,304	$ 735 - $ 1,872		Gamma	[36, 37]
Emergency Department Visit	$ 1,997	$ 1,498 - $ 2,497	(± 25%)	Gamma	[36]
HF Medications	$ 367	$ 275 - $ 459	(± 25%)	Gamma	[37]
Worsening HF Medications	$ 2,893	$ 1,357 - $ 4,430		Gamma	[38]
Post-Acute Care (Worsened HF)	$ 4,538	$ 1,521 - $ 7,852		Gamma	[38–40]

^a^Intervention cost estimates were based on implementation assumptions; see Additional File 1 (Tables S1, S2, & S3) for details

### Model parameters

Inputs for the decision tree model were derived from the parameters listed in Table 1. The text below summarizes how these parameters were identified, sources of their reported values, the methods used to assign them, and any conversions applied.

#### Clinical probabilities

Each cycle, patients would have good medication adherence (80% or higher) or poor adherence (less than 80%) that would then lead into clinical probabilities of the tree. The probability of having good or poor adherence was based on the clinical study’s data [25]. We used the study’s reported data and used an 80% adherence cutoff for before and after the intervention to estimate adherence for the usual care group and the intervention group. The 80% cutoff for medication adherence is widely used in research and clinical settings as a standard threshold to differentiate between good and poor adherence [41]. Importantly, it has been used in HF research and is related to hospitalization risk (see below).

Clinical probabilities, shown in Table 1, were based on published data that focus on HF outcomes in the US. Either of the pathways of adherence can lead to one of the two pathways: HF exacerbation or no HF exacerbation. For this analysis, a HF exacerbation was defined as the acute worsening onset of heart failure symptoms. Following a HF exacerbation, a person has the chance of dying within the year cycle or living. A person who lives from a HF exacerbation will transition into worsening HF symptoms or recover from the exacerbation and improve back to stable HF. A person who has no HF exacerbation can die from other causes or live while continuing to have HF. The clinical probabilities in this model were derived from studies that are closely related to the study population and outcomes of interest, seen in Table 1 and discussed in text below.

To estimate the probability of a HF hospitalization, we used data from Fitzgerald et al. (2011), which reported a hazard ratio (HR) of 1.81 of hospitalization for nonadherent patients (< 80% adherence) versus adherent patients. We calculated an annual probability of 23.1% for adherent patients and 41.8% for nonadherent ones (see Model Parameter Justification in Additional File 1). We used Solomon et al. (2007) to estimate living following hospitalization from a HF exacerbation. For patients who did not experience an exacerbation or an acute event, we estimated survival using data from Jones et al. (2019), which followed a large cohort of people living with chronic HF. The model’s post-hospitalization increased severity of HF was estimated from Khan et al. (2021) that looked at trends for readmission rates following a HF hospitalization. In most cases, we relied on single studies because no pooled estimates were available; the most relevant and recent data were chosen.

#### Health state utilities

Health state utility values were used in this study to describe the impact on someone’s health of conditions such as HF. These values range from 0 to 1, 0 representing death and 1 being full health [42]. Utility values were extracted from published estimates for HF exacerbation and hospitalization, stable HF, and NYHA classification. The mean value of the NYHA II-IV class utility were reflected in the worsening symptoms utility value. These values were then calculated into full path QALY totals based on clinical transitions in the decision tree.

To estimate total QALYs for each clinical pathway in the decision tree, the utility values were applied proportionally to the duration spent in each health state. Formulas were developed to account for time spent in death, exacerbation, recovery, or stable health. We assumed that people with HF who die do so halfway through the year (6 months) [43]. We also assumed that the average length of time that it takes for someone to recover from a HF exacerbation is one month, so all calculations account for a HF exacerbation utility value for one month out of the year. Following the one-month exacerbation, the rest of the year is based on the final clinical transition. These calculated full-path QALYs reflect the combined effects of utility and duration.

#### Costs

All costs were reported between in US dollars from 2015 to 2021 and were adjusted to 2025 values using the US Consumer Price Index (CPI) for All Urban Consumers [44]. Cost parameter inputs were derived and summarized from published literature on hospitalizations, outpatient visits, emergency department visits, HF medication, worsening HF medication, and post-acute care for worsening HF. Heart failure hospitalization costs reflected inpatient admissions for care related to heart failure only. Outpatient visit costs included both primary care appointments and specialty clinic encounters. Emergency department costs included acute, unscheduled care delivered exclusively in an emergency room setting. HF medication costs represented the annual cost of medications prescribed as part of routine HF management. Worsening HF medication costs included medications initiated or the dosage intensified in response to a HF event. Post-acute care costs comprised of services used after a worsening HF event, including long-term care facilities, rehabilitation, and home health services. The costs were calculated as the frequency of use yearly based on the clinical transition pathway from the decision tree. The intervention cost for the first year and subsequent year were estimated based on a structured budget informed by expert opinion and insights gained from implementation during the clinical study Hawkins & Firek (2014). The intervention activities and associated costs included clinical assessment, pharmacist or clinician visit for medication review, pictorial medication sheet creation and check, review of and update to medication sheet and quarterly pill counts as appropriate, software, and training for patient and staff.

### Sensitivity analyses

Sensitivity analyses examined uncertainty in key input parameters and the effects of that uncertainty on results. One-way deterministic analysis evaluated the effect of varying individual input parameters on the model’s base case outcome while holding other parameters constant. Inputs selected for this were those that were estimated based on little data, had substantial known uncertainty, or significantly influenced outcomes.

A probabilistic sensitivity analysis using Monte Carlo simulation was conducted to reflect uncertainty across all input parameters simultaneously. A total of 1000 iterations were run to simulate the range and distribution of the outputs.

Scenario analyses were conducted to explore the potential impact of changing input parameters to reflect the possible future implementation scenarios: richer-better, cheaper-less effective, and equity focused. In the extended two-year model, we examined three key clinical parameters under both high and low value assumptions: effectiveness, costs, and risk.

For the two-year model, assumptions were applied varying key input parameters from Table 1 to reflect potential changes in intervention effectiveness, costs, and clinical risks over time. Table 2 summarizes the parameter ranges used for each scenario specifically to adherence gains, HF exacerbation with good adherence, and subsequent-year intervention costs. These scenarios were designed to test potential future variations, such as improved or reduced intervention efficacy from gain in adherence, changes in costs over time (more or less expensive subsequent years), and differing risks of HF exacerbations among people who had good adherence in the intervention group. This allowed the model to explore mixed scenarios to assess the robustness of the cost-effectiveness of the intervention over a two-year horizon.

**Table 2:** Two-year model input assumptions for scenario analysis

Scenario assumptions	Gain in good adherence (Intervention)	HF Exacerbation with good adherence	Intervention subsequent year
Same Efficacy & Costs	Base Case	Base Case	Base Case
More Effective	Upper Bound	—	—
Less Expensive	—	—	Lower Bound
More Effective & Less Expensive	Upper Bound	—	Lower Bound
Less Effective	Lower Bound	—	—
More Expensive	—	—	Upper Bound
More Effective & Lower Risk	Upper Bound	Lower Bound	—
Higher Risk	—	Upper Bound	—

### Software

Data management and modeling were completed using Microsoft Excel for Mac, Version 16.98 (Microsoft 365 Subscription). Statistical analyses were conducted using a Visual Basic for Applications (VBA) macro in Microsoft Excel. The macro was designed to run probabilistic sensitivity analyses by randomly sampling input parameters’ ranges. In the Monte Carlo simulation, the macro sampled all input values and recalculated the decision tree model’s outputs, storing the results in an output sheet.

### Cost-effectiveness

The decision tree model calculated the change in costs and QALYs. The model produced a primary output known as an ICER; defined as the incremental cost per QALY gained for a patient living with HF who uses the pictorial medication sheet (and thus is more likely to achieve good adherence) compared to usual care. Costs and clinical events were not discounted due to the short time-horizon of the model.

## Results

### Cost-effectiveness compared to usual care

In the on-year base case analysis, patients in the intervention group had an average cost of $22,557 compared to $24,507 with usual care, saving $1,949 per patient. Implementation for 100 individuals would yield 0.45 QALYs and save $195 thousand.

The lower cost reflects savings in medical care higher than intervention costs. They gained 0.691 QALYs versus 0.686 with usual care, an increase of 0.0045. Thus, over the course of one year, the pictorial medication sheet was “dominant”: less costly and more effective compared to usual care. With dominance, and thus no tradeoff between costs and efficacy, no ICER is needed or appropriate.

### Sensitivity analyses

The following subsections present the results from four types of sensitivity analyses performed in this study.

#### Deterministic one-way sensitivity analysis

**Fig. 3:**
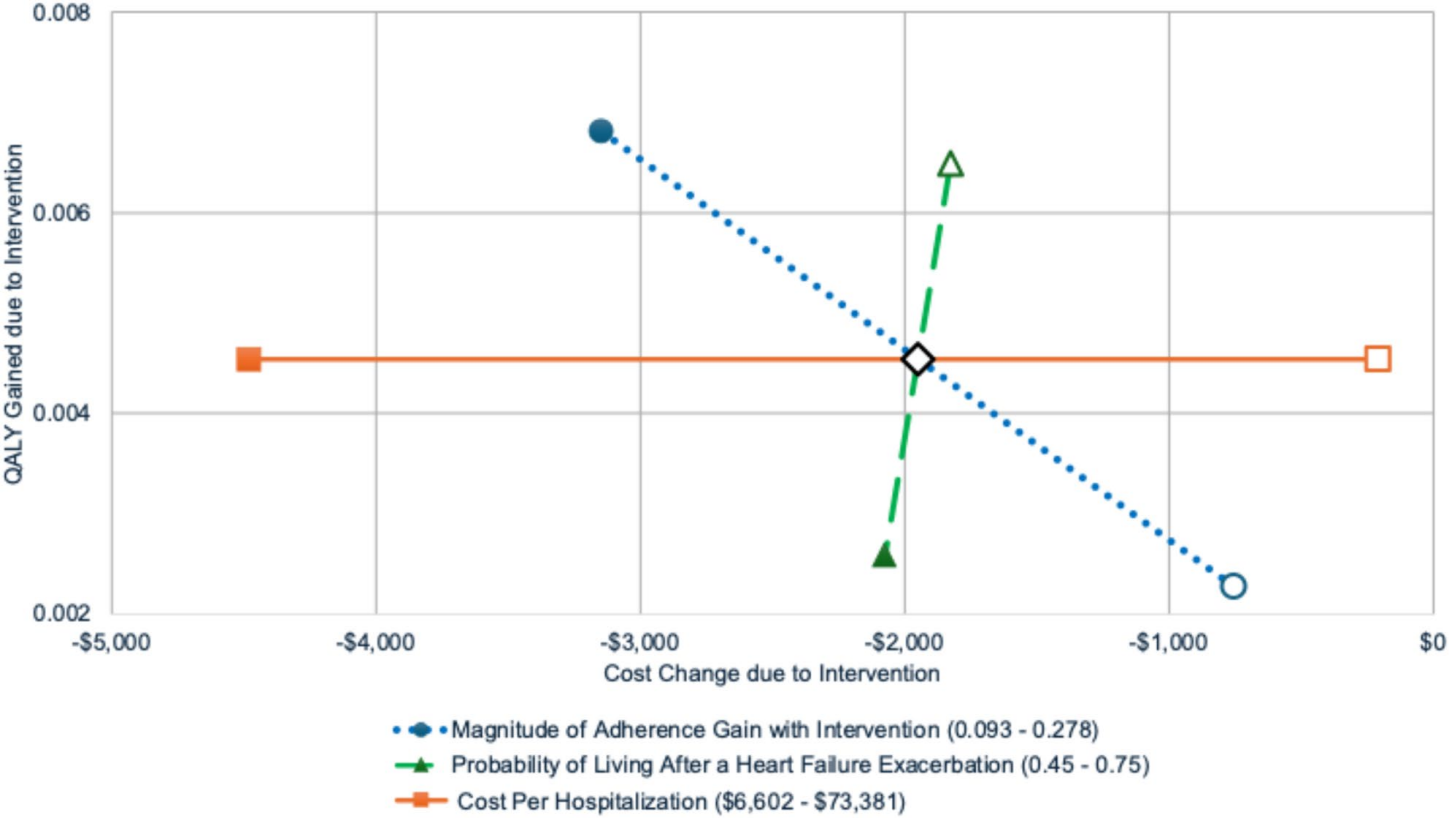
Deterministic one-way sensitivity analyses for three key inputs. Each line represents one input varied across its uncertainty range while holding other inputs constant. Open markers are lower bound values and solid markers upper bound; values are reported in the key. The central diamond is the base case result. Uncertainty in cost per hospitalization (solid orange line) varies the predicted cost change from -$4,479 (at the upper bound) to -$206, with no effect on QALYs. A lower adherence gains from the intervention (dotted blue line) results in lower savings and QALY gains, and vice-versa. A higher probability of living after a HF exacerbation (dashed green line) shifts the results toward lower QALY gains and higher cost savings

Deterministic one-way sensitivity analysis was done for all model input parameter values to assess the impact of uncertainties on the incremental cost and QALY outcomes of the intervention compared to usual care. While the full analysis tested variation across all parameters, three influential inputs are highlighted here: the magnitude of adherence gain achieved with the intervention, the probability of surviving after a HF exacerbation with good adherence, and the cost per hospitalization event. Figure 3 illustrates how variation in key parameters influences the cost and QALY difference between the intervention and usual care. For all three inputs, the intervention remains dominant: it gains QALYs and lowers costs across all values. See detailed explanation in the Fig. 3 legend.

#### Probabilistic sensitivity analysis

**Fig. 4:**
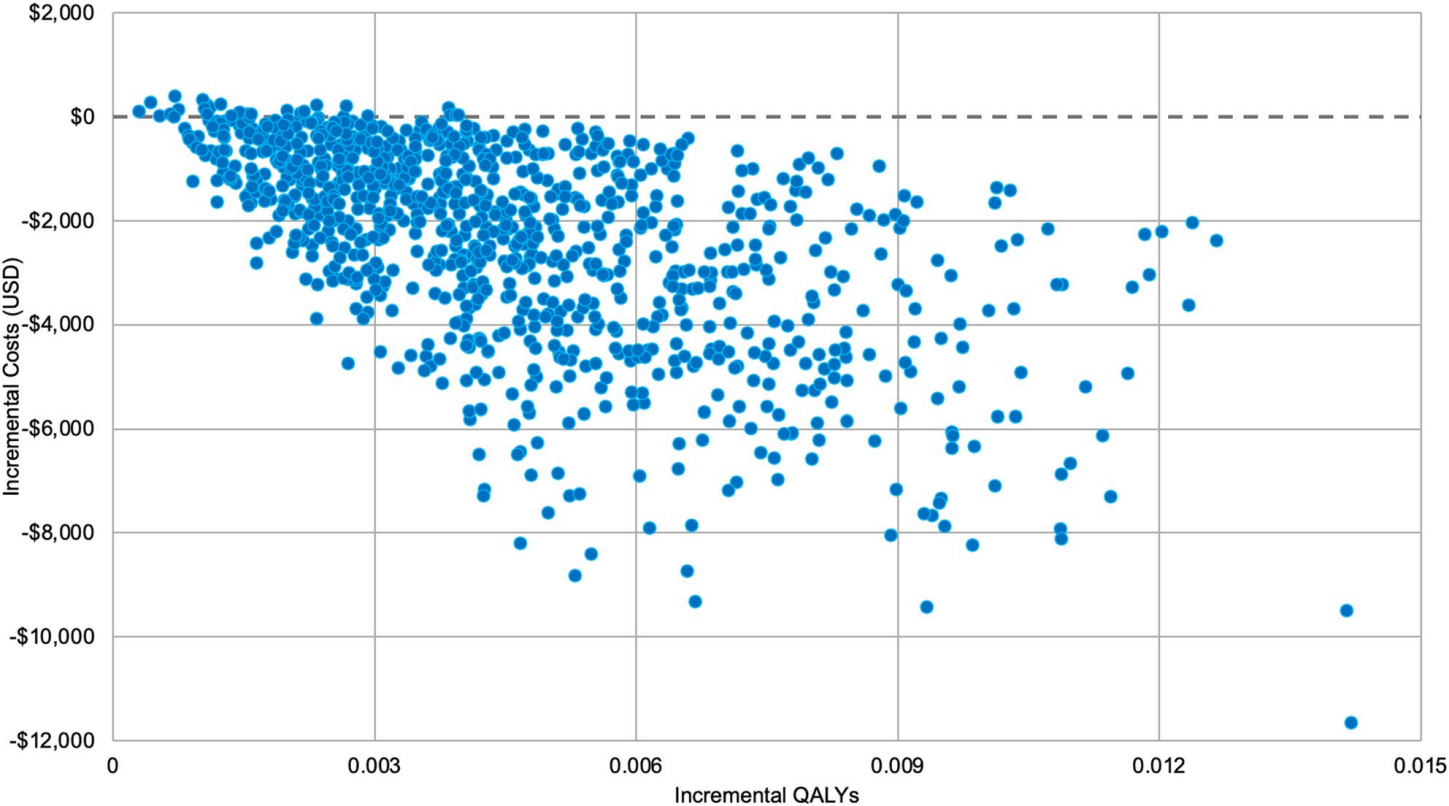
Probabilistic sensitivity analysis: Monte carlo simulation. Each blue dot in the figure represents a simulated outcome for the one-year model, illustrating the range of possible changes in costs and QALYs based on the parameter ranges for uncertainty

A probabilistic sensitivity analysis for the 1-year analysis is presented in Fig. 4 with 1,000 iterations to evaluate joint parameter uncertainty. The incremental cost ranged from negative $11,651 to $403, with a gain in QALYs from 0.0003 to 0.0142. Results showed that 97.3% of simulations fell in the lower right quadrant (dominant), indicating cost savings with improved health outcomes compared to usual care. The remaining 2.7% of iterations fell in the upper right quadrant, representing positive incremental costs with QALY gains. Among the iterations with positive incremental costs, ICER values ranged from $2,782 to $628,944. Out of the total of 27 ICERs, 20 were below a willingness-to-pay threshold of $150,000 per QALY. Overall, the probabilistic sensitivity analysis demonstrated that 99.3% of the iterations from the simulation are dominant or cost-effective.

#### Scenario analysis

Scenario analysis was performed to evaluate the impact of alternative program designs on the cost-effectiveness outcomes compared to the base-case parameter values (Table 3). The richer-better program scenario assumes a comprehensive, well-funded program with upper-bound intervention costs and high adherence gains; the intervention is dominant with a cost savings of $3,034 and a QALY gain of 0.009. The cheaper-less effective program scenario assumes a reduced-price rollout with lower adherence gains. The intervention was dominant with a cost savings of $932 and a gain in QALY of 0.003. The equity-focused program scenario assumes a more targeted, culturally tailored program with higher costs for services such as translation and base-case adherence gains. The intervention remained dominant with a cost savings of $1,702 and a gain in QALY of 0.006.

**Table 3:** One-year scenario analysis results

Inputs changed	Change from base-case	∆ Costs	∆ QALYs	ICER
Base Case		-$1,949	0.0045	^a^Dominant
Richer-Better Program				
Intervention Cost	Upper Bound (+ $124)	-$3,034	0.0068	Dominant
Gain in Good Adherence (Intervention)	Upper Bound (+ 0.093)			
Cheaper-Less Effective Program				
Intervention Cost	Reduced Base Case (- $200.05)	-$932	0.0023	Dominant
Gain in Good Adherence (Intervention)	Lower Bound (− 0.093)			
Equity-Focused Program				
Intervention Cost	Increased Base Case (+ $279)	-$1,702	0.0045	Dominant
Good Adherence (Usual Care)	Lower Bound (− 0.139)			
Gain in Good Adherence (Intervention)	Base Case (0.000)			

^a^Dominant indicates the intervention is less costly and more effective than usual care

#### Two-year model sensitivity analysis

Table 4 summarizes the two-year sensitivity analysis results across varying scenarios. In all cases, the intervention remained the dominant strategy. Base case results from the one-year model were included in the table for comparison. The incremental QALYs for all scenarios at least doubled in the two-year model. This reflects more patients in the intervention arm surviving the first year and thus accumulating many more added QALYs from the start of Year 2. Scenarios with greater effectiveness, lower costs, reduced risk, or a combination of these three produced the largest improvements in cost-savings and QALYs gained.

**Table 4:** Two-year sensitivty analysis results under year-2 assumptions

Scenario	∆ Costs	∆ QALYs	ICER
Base Case Year 1	-$1,949	0.00454	^a^Dominant
Two-Year Results			
Same Efficacy & Costs in Y2	-$3,031	0.02917	Dominant
More Effective in Y2	-$3,784	0.03108	Dominant
Less Expensive in Y2	-$3,102	0.02917	Dominant
More Effective & Less Expensive in Y2	-$3,855	0.03108	Dominant
Less Effective in Y2	-$2,279	0.02725	Dominant
More Expensive in Y2	-$2,961	0.02917	Dominant
More Effective and Lower Risk in Y2	-$4,497	0.03220	Dominant
Higher Risk in Y2	-$2,550	0.02864	Dominant

^a^Dominant indicates the intervention is less costly and more effective than usual care

## Discussion

In this evaluation, we found that the pictorial medication sheet intervention dominates over usual care, meaning it is cost saving and improves health outcomes measured by QALYs. This dominant result was influenced primarily by rates of good medication adherence in the intervention arm, reduced risk of exacerbations associated with better adherence, thus lower hospitalization costs, and low cost of implementing the intervention. Overall, patients receiving the intervention were more likely to remain stable or return to their baseline health status, reducing high costs of hospitalizations and post-acute care. These findings were found to be consistent with existing literature the also found small improvements in medication adherence can influence lower costs [45, 46]. Although there is limited recent evidence evaluating pictorial medication sheets or similar tools for medication adherence among patients with HF and cognitive impairment, more recent work supports the use of patient-held medication lists to improve adherence and medication safety [47, 48]. Despite the supportive clinical evidence, there remains the lack of economic evaluation for simple and visually supportive medication tools tailored to older adults with chronic conditions and cognitive impairment. Overall, from an economic and practical perspective, implementing a simple, low-cost intervention can have a large impact on healthcare spending and patient health.

This analysis could also have important policy implications. The intervention aligns well with the objectives of the Centers for Medicare and Medicaid Services (CMS), specifically the CMS Innovation Center. The Innovation Center’s mission is to test and scale new service delivery models that improve care quality while reducing costs [49]. By modeling the cost-effectiveness of the pictorial medication sheet, our analysis provides evidence that can help support future CMS Innovation Center initiatives and could be adopted into broader insurance coverage models. Designing policies that incentivize or reimburse such a low-cost and high-value intervention can help drives its implementation at scale, overall improving health outcomes for older adults in the US. Although QALYs gained in this analysis were small, improvements in medication adherence may still reduce preventable hospitalizations and costs when implemented at scale. This makes the pictorial medication sheet well suited for CMS Innovation Center’s models that test real-world impact among older adults managing complex medication regimens.

The study has several limitations. The clinical study evaluating the pictorial medication sheet had a small sample size (*n* = 27) and primarily conducted among male veterans, which could limit its generalizability. Additionally, the study lacked a randomized control group, requiring the use of pre-intervention adherence data to represent usual care. There were also limitations structural limitations in the decision tree model. The model assumed that the population was well-controlled for HF and other health related factors, which may not reflect real-world heterogeneity. The one-year model also used adherence rates observed over only four-months in the clinical study, which may not accurately capture longer-term adherence behavior. Another limitation was finite amount of clinical outcome probabilities and utility values feeding into the model, limiting the availability of robust data, meaning the model might not fully reflect real-word event probabilities or patient quality of life trajectories. Finally, the simplicity of the decision tree model limits its ability to account for the full range of variability in health outcomes associated with HF and polypharmacy in older adults living with cognitive impairment.

Future research should address these limitations. From a modeling perspective, the development of a longer time horizon could better capture the full HF progression, survival, costs, and quality of life impacts. These future models could more evaluate the value of improvement in medication adherence long-term, although longer time frames also increase uncertainty. Empirical research should also prioritize larger, more diverse study populations and randomized control trials with longer follow-up periods to measure medication adherence over time for simple, low-cost visual aids.

The study population for the pictorial medication sheet was specifically designed for people living with cognitive impairment and HF, but its potential benefits may extend beyond this population. Policymakers and healthcare stakeholders should consider if the intervention applies more broadly across the geriatric population; after all, medication does not work if it is not taken. This simple statement reflects the somber truth of the increased costs in terms of outcomes, quality of life, and health care dollars when patients do not take their prescribed medications. Heart failure is only one of many prevalent chronic conditions in this population with guideline-based complex medication regimens; others include diabetes mellitus, hypertension, depression, and chronic obstructive pulmonary disease [50].

We further propose that this intervention should not be limited to those with cognitive impairment. About 43% of older adults in the US take five or more prescription medications, suggesting that the pictorial medication sheet can reduce healthcare costs and improve quality of life for nearly half of this population [51]. Visual aids have been shown to improve medication adherence across a wide range of patient populations by supporting health literacy, and may offer an attractive method to improve adherence for persons who are not English language proficient [52, 53]. Health systems should try to seek low-cost, scalable strategies to improve medication adherence and reduce avoidable hospitalizations, interventions like the pictorial medication sheet provide a potential promising and practical solution that can support older adults in the US.

## Conclusion

The pictorial medication sheet in this study indicated an improvement in quality of life and lowered healthcare costs related to HF for older adults living with cognitive impairment. These findings show the potential for a simple, but low-costing intervention to increase good medication adherence and reduce high healthcare costs.

## Supplementary Information

Below is the link to the electronic supplementary material.

Supplementary Material 1

## Data Availability

No new datasets were generated or analyzed in this study. All data inputs and parameters are derived from previously published sources that are referenced in the manuscript. The decision tree model was developed in Microsoft Excel and is available from the corresponding author upon request.

## Abbreviations

HF: Heart Failure
QALY: Quality-Adjusted Life Year
HR: Hazard Ratio
CPI: Consumer Price Index
VBA: Visual Basic for Applications
CMS: Centers for Medicare and Medicaid Services
