# Supplementary material for: Cost-effectiveness of a pictorial medication sheet for older adults with heart failure and cognitive impairment
Source: BMC Health Serv Res. 2026 Jan 21;26:247. doi: 10.1186/s12913-026-14072-6 (PMC12905874; doi:10.1186/s12913-026-14072-6)
Supplement: Supplementary file 1 — Supplementary Material 1 [file 12913_2026_14072_MOESM1_ESM.docx]

Supplementary Material

Cost-Effectiveness of Pictorial Medication Sheet to Increase Adherence in Older Adults with Heart Failure and Cognitive Impairment

Author(s):

Kaylee B Vannoy^1^, Lee Ann Hawkins^2^, James G Kahn^3^

Affiliation(s):

^1^ University of California, San Francisco, Institute for Global Health Sciences, San Francisco, CA, USA

^2^ Indiana Wesleyan University, Graduate Nursing Division, Marion, IN, USA

^3^ University of California, San Francisco, Philip R Lee Institute for Health Policy Studies, San Francisco, CA, USA

Corresponding author:

Kaylee Vannoy, Email: [kaylee.vannoy@ucsf.edu](mailto:kaylee.vannoy@ucsf.edu)

Table S1. Initial Personnel Costs to Implement Intervention

Table S2. Recurring Personnel Costs to Implement Intervention

Table S3. Non-Personnel Costs to Implement Intervention

Table S4. Cost Sheet Review

Table S5. Path QALY (calculated)

Table S6. Probabilistic Sensitivty Analysis: ICERs

Table S7. CHEERS Checklist

Model Parameter Justification

References

**Table S1.** Initial Personnel Costs to Implement Intervention

| **Step** | **Summary** | **Who is involved** | **Annual Salary + Benefits** | **Hourly Rate** | **Estimated time (min)** | **Cost** | **Reference** |
| --- | --- | --- | --- | --- | --- | --- | --- |
| **1** | Initial Primary Care Visit: Discuss medication management and assess cognitive function and health literacy. | Nurse | $86,070 | $43.04 | 30 | $21.52 | [1] |
| **2** | Explain the pictorial medication sheet and its benefits by being part of the program. | Primary Care Provider | $277,000 | $138.50 | 5 | $11.54 | [2] |
| **3** | Obtain consent to participate and address any concerns. | Nurse | $86,070 | $43.04 | 5 | $3.59 | [1] |
| **4** | Conduct a medication review and verify medications with medical records; adjust if needed. | Pharmacist | $136,030 | $68.02 | 30 | $34.01 | [3] |
| **5** | Sheet creation: Find medication images, design, and print the pictorial medication sheet. | Pharmacy Technician | $40,300 | $20.15 | 20 | $6.72 | [4] |
| **6** | Translation of pictorial medication sheet into patient's preferred language (if needed; assume 25%) | Translator | $57,090 | $28.55 | 15 | $7.14 | [5] |
| **7** | Check medication sheet for accuracy and give and walk through the sheet with patient or caregiver. | Pharmacist | $136,030 | $68.02 | 10 | $11.34 | [3] |

**Table S2.** Recurring Personnel Costs to Implement Intervention

| **Step** | **Summary** | **Who is involved** | **Annual Salary + Benefits** | **Hourly Rate** | **Estimated time (min)** | **Cost Monthly** | **Reference** |
| --- | --- | --- | --- | --- | --- | --- | --- |
| **1** | Pill counts (quarterly) | Pharmacy Technician | $40,300 | $20.15 | 15 | $5.04 | [4] |
| **2** | Brief medication check-in through routine visit or phone call (monthly) | Nurse | $86,070 | $43.04 | 10 | $7.17 | [1] |
| **3** | Update medications and adjust sheet (quarterly) | Pharmacist | $136,030 | $68.02 | 6.67 | $7.56 | [3] |
| **4** | Re-design and print updated sheet (quarterly) | Pharmacy Technician | $40,300 | $20.15 | 6.67 | $2.24 | [4] |
| **5** | Review sheet with patient or caregiver | Pharmacist | $136,030 | $68.02 | 10 | $11.34 | [3] |

**Table S3.** Non-Personnel Costs to Implement Intervention

| **Non-personnel items** | **Input unit** | **# Needed** | **Price** | **Cost** | **Reference** |
| --- | --- | --- | --- | --- | --- |
| **Supplies** |  |  |  |  |  |
| Pictorial medication sheet – plastic sheet protectors | sheet | 1.00 | $0.20 | $0.20 | [6] |
| Cost per page = catridge price / page yield + paper cost | sheet | 1.00 | $0.10 | $0.10 | [7] |
|  |  |  |  |  |  |
| **Equipment** |  |  |  |  |  |
| Software – WedMD pill identifier | annual | 0.002 | $0 | $0.00 | [8] |
|  |  |  |  |  |  |
| **Services** |  |  |  |  |  |
| Surcharge (utilities, cleaning, rent) | month | Mothly cost share | $0 | $0.00 | NA |
| Graphic design support | one time cost | 500.00 | $500.00 | $1.00 | [9] |
|  |  |  |  |  |  |
| **Space** |  |  |  |  |  |
| Rent (for developing and storing materials) | month | Per month facility rent | $0 | $0 | NA |
|  |  |  |  |  |  |
| **Staff Training** |  |  |  |  |  |
| Bi-annual 2 days | Per 500 patients | 0.03 | $20.15 | $0.64 | [3] |

**Table S4.** Cost Sheet Review

| **Cost Type** | **Mean Cost** | **Year of Cost Estimate** | **Where** | [**CPI (2025)**](https://data.bls.gov/timeseries/CUUR0000SA0) | **Reference** |
| --- | --- | --- | --- | --- | --- |
| Total first year cost of Intervention | $497.89 | 2025 | US | $497.89 | See Tables S1, S2, S3 |
| Subsequent year total cost of intervention | $400.10 | 2025 | US | $400.10 | See Table S2 |
| All cause hospitalization cost over 1 year | $26,998.00 | 2019 | US | $33,722.67 | [10] |
| HF-specific hospitalization cost over 1 year | $19,907.00 | 2019 | US | $24,865.44 | [10] |
| HF-specific hospitalization cost | $14,323.00 | 2019 | US | $17,890.58 | [10] |
| Cost per ED services | $1,599.00 | 2019 | US | $1,997.28 | [10] |
| Cost per outpatient visit | $1,499.00 | 2019 | US | $1,872.37 | [10] |
| Inpatient costs post index hospitalization | $41,750.00 | 2015 | US | $56,250.43 | [11] |
| Outpatient costs post index hospitalization | $5,828.00 | 2015 | US | $7,852.16 | [11] |
| Total index hospitalization | $10,500.20 | 2018 | US | $13,353.27 | [12] |
| Total 90-day post-acute care (includes outpatient) | $2,525.84 | 2018 | US | $3,212.15 | [12] |
| Total 30-day post-acute care (includes outpatient) | $2,743.80 | 2018 | US | $3,489.33 | [12] |
| All outpatient prescriptions 12 months following a worsening HF event | $3,288.00 | 2015 | US | $4,429.97 | [13] |
| HF related medications 12 months following a worsening HF event | $1,007.00 | 2015 | US | $1,356.75 | [13] |
| All outpatient encounters outpatient encounters 12 months following a worsening HF event | $4,910.00 | 2015 | US | $6,615.32 | [13] |
| HF related outpatient encounters 12 months following a worsening HF event | $ 1,129.00 | 2015 | US | $ 1,521.12 | [13] |
| All hospital encounters 12 months following a worsening HF event | $ 54,465.00 | 2015 | US | $ 73,381.56 | [13] |
| HF-related hospital encounters 12 months following a worsening HF event | $ 33,206.00 | 2015 | US | $ 44,738.97 | [13] |
| HF hospitalization annual cost | $ 5,602.51 | 2021 | US | $ 6,602.54 | [14] |
| HF routine medication annual cost | $ 311.66 | 2021 | US | $ 367.29 | [14] |
| HF outpatient annual cost | $ 623.71 | 2021 | US | $ 735.04 | [14] |

**Table S5.** Path QALYs (calculated)

| **Full Path QALY Inputs** | **Base Case** | **Lower Bound** | **Upper Bound** | **Distribution** | **Reference** |
| --- | --- | --- | --- | --- | --- |
| HF Exacerbation → Die | 0.372 | 0.369 | 0.374 | Beta | [15] |
| HF Exacerbation → Live → Worsened HF | 0.672 | 0.652 | 0.692 | Beta | [15,16] |
| HF Exacerbation → Live → Recovered | 0.762 | 0.757 | 0.766 | Beta | [15] |
| No HF Exacerbation → Die | 0.390 | 0.388 | 0.392 | Beta | [15] |
| No HF Exacerbation → Live | 0.780 | 0.775 | 0.785 | Beta | [15] |

**Table S6.** Probabilistic Sensitivty Analysis: ICERs

| **Delta QALYs** | **Delta Costs** | **ICER** |
| --- | --- | --- |
| 0.0007 | $2 | $2,782 |
| 0.0029 | $20 | $6,690 |
| 0.0040 | $33 | $8,386 |
| 0.0039 | $39 | $10,055 |
| 0.0039 | $40 | $10,460 |
| 0.0014 | $16 | $11,663 |
| 0.0005 | $16 | $30,394 |
| 0.0015 | $47 | $30,698 |
| 0.0016 | $57 | $35,989 |
| 0.0022 | $78 | $36,034 |
| 0.0039 | $170 | $44,055 |
| 0.0011 | $51 | $47,319 |
| 0.0022 | $110 | $50,363 |
| 0.0015 | $80 | $55,379 |
| 0.0020 | $114 | $56,917 |
| 0.0027 | $213 | $79,540 |
| 0.0007 | $58 | $87,810 |
| 0.0023 | $234 | $99,875 |
| 0.0011 | $156 | $148,164 |
| 0.0011 | $167 | $149,795 |
| 0.0012 | $236 | $191,680 |
| 0.0007 | $147 | $196,895 |
| 0.0011 | $228 | $210,568 |
| 0.0010 | $331 | $323,591 |
| 0.0003 | $102 | $338,728 |
| 0.0007 | $403 | $565,758 |
| 0.0004 | $270 | $628,944 |

**Table S7.** CHEERS Checklist [17]

| **Section/topic** | **Item no** | **Guidance for reporting** | **Reported in section** |
| --- | --- | --- | --- |
| Title | | | |
| Title | 1 | Identify the study as an economic evaluation and specify the interventions being compared | Title Page |
| Abstract | | | |
| Abstract | 2 | Provide a structured summary that highlights context, key methods, results, and alternative analyses | Page 1 |
| Introduction | | |  |
| Background and objectives | 3 | Give the context for the study, the study question, and its practical relevance for decision making in policy or practice | Pages 1-2 |
| Methods | | | |
| Health economic analysis plan | 4 | Indicate whether a health economic analysis plan was developed and where available | Pages 2-8 |
| Study population | 5 | Describe characteristics of the study population (such as age range, demographics, socioeconomic, or clinical characteristics) | Pages 2-3 |
| Setting and location | 6 | Provide relevant contextual information that may influence findings | Page 2 |
| Comparators | 7 | Describe the interventions or strategies being compared and why chosen | Page 4 |
| Perspective | 8 | State the perspective(s) adopted by the study and why chosen | Page 3 |
| Time horizon | 9 | State the time horizon for the study and why appropriate | Page 4 |
| Discount rate | 10 | Report the discount rate(s) and reason chosen | Page 8 |
| Selection of outcomes | 11 | Describe what outcomes were used as the measure(s) of benefit(s) and harm(s) | Page 8 |
| Measurement of outcomes | 12 | Describe how outcomes used to capture benefit(s) and harm(s) were measured | Pages 4-5 |
| Valuation of outcomes | 13 | Describe the population and methods used to measure and value outcomes | Pages 5-7 |
| Measurement and valuation of resources and costs | 14 | Describe how costs were valued | Page 6 |
| Currency, price date, and conversion | 15 | Report the dates of the estimated resource quantities and unit costs, plus the currency and year of conversion | Page 6 |
| Rationale and description of model | 16 | If modelling is used, describe in detail and why used. Report if the model is publicly available and where it can be accessed | Pages 4-5 |
| Analytics and assumptions | 17 | Describe any methods for analyzing or statistically transforming data, any extrapolation methods, and approaches for validating any model used | Pages 4-6 |
| Characterizing heterogeneity | 18 | Describe any methods used for estimating how the results of the study vary for subgroups | Page 12 |
| Characterizing distributional effects | 19 | Describe how impacts are distributed across different individuals or adjustments made to reflect priority populations | Pages 4-6 |
| Characterizing uncertainty | 20 | Describe methods to characterize any sources of uncertainty in the analysis | Pages 7-8 |
| Approach to engagement with patients and others affected by the study | 21 | Describe any approaches to engage patients or service recipients, the general public, communities, or stakeholders (such as clinicians or payers) in the design of the study | Pages 4-3 and 12 |
| Results | | |  |
| Study parameters | 22 | Report all analytic inputs (such as values, ranges, references) including uncertainty or distributional assumptions | Pages 6-8 and 10-11 |
| Summary of main results | 23 | Report the mean values for the main categories of costs and outcomes of interest and summarize them in the most appropriate overall measure | Page 8 |
| Effect of uncertainty | 24 | Describe how uncertainty about analytic judgments, inputs, or projections affect findings. Report the effect of choice of discount rate and time horizon, if applicable | Pages 8-11 |
| Effect of engagement with patients and others affected by the study | 25 | Report on any difference patient/service recipient, general public, community, or stakeholder involvement made to the approach or findings of the study | Page 12 |
| Discussion | | |  |
| Study findings, limitations, generalizability, and current knowledge | 26 | Report key findings, limitations, ethical or equity considerations not captured, and how these could affect patients, policy, or practice | Pages 12-13 |
| Other relevant information | | | |
| Source of funding | 27 | Describe how the study was funded and any role of the funder in the identification, design, conduct, and reporting of the analysis | Page 13 |
| Conflicts of interest | 28 | Report authors conflicts of interest according to journal or International Committee of Medical Journal Editors requirements | Page 13 |

**Model Parameter Justifications**

*Clinical Probabilities*

The model distinguished between patients who adhered to heart failure medications and who did not. Adherence was defined as taking at least 80% of the prescribed medications in the intervention’s study [18]. Adherence rates for the usual care group were derived from pre-intervention data, while post-intervention results were when the intervention was used by patients. To estimate the probability of heart failure exacerbation, a study reported a hazard ratio of 1.81 for hospitalization among nonadherent compared to adherent patients [19]. We used the rate of 143 hospitalizations among 557 patients, then we solved for the probability of hospitalization among adherent patients and yielded an annual hospitalization probability of 23.2% for adherent patients and 41.9% for nonadherent patients. Mortality risk was stratified by clinical status. Patients hospitalized with exacerbation/decompensation, one study found 1,455 patients were admitted with heart failure and 869 survived to the end of the study period [20]. Among patients with stable heart failure, one-year survival was taken as 86.5% [21]. The probability of worsening heart failure following an exacerbation was linked to 90-day readmission risk and reported as 15% [22].

*Quality-Adjusted Life Years (QALYs)*

Utility values were used to estimate quality of life across modeled states. Patients experiencing exacerbation who also died were assigned baseline and exacerbation utility values, reflecting an average one-month decline followed by death occurring midway through the annual cycle. Survivors of exacerbation with worsening heart failure were modeled with one month of reduced utility during an acute event, followed by six months at lower values consistent with the New York Heart Association class II-IV symptoms. Patients who recovered to baseline after exacerbation experienced the same initial one-month decline but then returned to near-baseline utility value for the following six months. For individuals with stable heart failure who died during the year, utility value was applied for six months at the stable baseline value, reflecting death halfway through the cycle. Patients with stable heart failure who remained alive retained baseline utility through the year duration.

*Cost Inputs*

Intervention implementation cost was applied to all patients who survived the entire one-year cycle, with half-year costs applied to those who died earlier. Hospitalization costs reflected the clinical course following an exacerbation. Patients who died during an exacerbation were assumed to have at least one admission. Survivors with worsening heart failure were assumed to experience one index hospitalization, one subsequent admission within six months, and a 50% probability of readmission within the following six months. Patients who returned to baseline had fewer admissions, limiting to one index event and a single follow-up. Outpatient visits varied by clinical course. Stable patients generally required 2-4 visits annually, whereas decompensated heart failure was associated with 6-12 visits per year. Survivors from an exacerbation with prior worsening heart failure were assumed to have nine visits per year, while those returning to baseline had six. Patients who died after exacerbation were modeled with two visits following hospitalization, while stable patients who died mid-year had one visit. Emergency department visits were assigned one or two per year among patients with recurrent exacerbations, and one or none for stable patients. All patients were charged for annual heart failure medications unless they died within the year, in which costs were prorated to six months. Patients surviving exacerbations were assumed to continue a more intense heart failure medication regimen, and those who died after an event accrued only one month of additional medication. Post-acute care costs were used for patients with severe heart failure following hospitalization. Patients who died after exacerbation were assumed to require one month of post-acute care, while those with worsened heart failure had six months of services. Patients who returned to baseline were assumed to require two months of post-acute care.

**References**

[1] Registered Nurses. Bur Labor Stat n.d. https://www.bls.gov/ooh/healthcare/registered-nurses.htm (accessed September 2, 2025).

[2] Lee M. Physician salary report 2025: Modest increase in compensation. Weatherby 2025. https://weatherbyhealthcare.com/blog/annual-physician-salary-report (accessed September 2, 2025).

[3] Pharmacists. Bur Labor Stat n.d. https://www.bls.gov/ooh/healthcare/pharmacists.htm (accessed September 2, 2025).

[4] Pharmacy Technicians. Bur Labor Stat n.d. https://www.bls.gov/ooh/healthcare/pharmacy-technicians.htm (accessed September 2, 2025).

[5] Interpreters and Translators. Bur Labor Stat n.d. https://www.bls.gov/ooh/media-and-communication/interpreters-and-translators.htm (accessed September 2, 2025).

[6] Sheet Protectors | Staples n.d. https://www.staples.com/sheet-protectors/cat_CL350730 (accessed September 2, 2025).

[7] Printing Costs: How To Accurately Calculate Your Printing Cost Per Page. Toner Buzz n.d. https://www.tonerbuzz.com/blog/printing-costs/ (accessed September 2, 2025).

[8] Pill Identification Tool. WebMD n.d. https://www.webmd.com/pill-identification/default.htm (accessed September 2, 2025).

[9] Flyer Design Rates You Need to Know About in 2024 n.d. https://servicelist.io/article/flyer-design-rates (accessed September 2, 2025).

[10] Urbich M, Globe G, Pantiri K, et al. A Systematic Review of Medical Costs Associated with Heart Failure in the USA (2014–2020). Pharmacoeconomics 2020;38:1219–36. https://doi.org/10.1007/s40273-020-00952-0.

[11] Olchanski N, Vest AR, Cohen JT, et al. Two-year outcomes and cost for heart failure patients following discharge from the hospital after an acute heart failure admission. Int J Cardiol 2020;307:109–13. https://doi.org/10.1016/j.ijcard.2019.10.033.

[12] Reinhardt SW, Clark KAA, Xin X, et al. Thirty-Day and 90-Day Episode of Care Spending Following Heart Failure Hospitalization Among Medicare Beneficiaries. Circ Cardiovasc Qual Outcomes 2022;15:e008069. https://doi.org/10.1161/CIRCOUTCOMES.121.008069.

[13] Givertz MM, Yang M, Hess GP, et al. Resource utilization and costs among patients with heart failure with reduced ejection fraction following a worsening heart failure event. ESC Heart Fail 2021;8:1915–23. https://doi.org/10.1002/ehf2.13155.

[14] Zheng J, Abudayyeh I, Rakovski C, et al. Trends in heart failure costs for commercially insured patients in the United States (2006–2021). BMC Health Serv Res 2024;24:780. https://doi.org/10.1186/s12913-024-11240-4.

[15] Ambrosy AP, Hernandez AF, Armstrong PW, et al. The clinical course of health status and association with outcomes in patients hospitalized for heart failure: insights from ASCEND-HF. Eur J Heart Fail 2016;18:306–13. https://doi.org/10.1002/ejhf.420.

[16] BrJCardiol. Impact of HF on HRQoL in patients and their caregivers in England: results from the ASSESS study - The British Journal of Cardiology n.d. https://bjcardio.co.uk/2017/03/impact-of-hf-on-hrqol-in-patients-and-their-caregivers-in-england-results-from-the-assess-study/ (accessed July 11, 2025).

[17] Husereau D, Drummond M, Augustovski F, et al. Consolidated Health Economic Evaluation Reporting Standards 2022 (CHEERS 2022) statement: updated reporting guidance for health economic evaluations. BJOG Int J Obstet Gynaecol 2022;129:336–44. https://doi.org/10.1111/1471-0528.17012.

[18] Hawkins LA, Firek CJ. Testing a novel pictorial medication sheet to improve adherence in veterans with heart failure and cognitive impairment. Heart Lung J Crit Care 2014;43:486–93. https://doi.org/10.1016/j.hrtlng.2014.05.003.

[19] Fitzgerald AA, Powers JD, Ho PM, et al. Impact of Medication Nonadherence on Hospitalizations and Mortality in Heart Failure. J Card Fail 2011;17:664–9. https://doi.org/10.1016/j.cardfail.2011.04.011.

[20] Solomon SD, Dobson J, Pocock S, et al. Influence of Nonfatal Hospitalization for Heart Failure on Subsequent Mortality in Patients With Chronic Heart Failure. Circulation 2007;116:1482–7. https://doi.org/10.1161/CIRCULATIONAHA.107.696906.

[21] Jones NR, Roalfe AK, Adoki I, et al. Survival of patients with chronic heart failure in the community: a systematic review and meta-analysis. Eur J Heart Fail 2019;21:1306–25. https://doi.org/10.1002/ejhf.1594.

[22] Khan MS, Sreenivasan J, Lateef N, et al. Trends in 30- and 90-Day Readmission Rates for Heart Failure. Circ Heart Fail 2021;14:e008335. https://doi.org/10.1161/CIRCHEARTFAILURE.121.008335.
